# Characterization and phylogenetic analysis of the complete mitochondrial genome of *Neurothemis fulvia* (Odonata: Anisoptera: Libellulidae)

**DOI:** 10.1080/23802359.2021.1875924

**Published:** 2021-02-17

**Authors:** Xinyuan Peng, Yuxia Gao, Xiang Song, Yimin Du

**Affiliations:** a School of Life Sciences, Gannan Normal University, Ganzhou, PR China ;; b National Navel Orange Engineering and Technology Research Center, Ganzhou, PR China

**Keywords:** Odonata, Libellulidae, mitochondrial genome, *Neurothemis fulvia*, phylogenetic analysis

## Abstract

*Neurothemis fulvia* is a dragonfly of wet forests and usually perches on fallen logs and shrubs. In this study, we sequenced and analyzed the complete mitochondrial genome (mitogenome) of *N. fulvia*. This mitogenome was 15,459 bp long and encoded 13 protein-coding genes (PCGs), 22 transfer RNA genes (tRNAs), and 2 ribosomal RNA unit genes (rRNAs). The nucleotide composition of the mitogenome was biased toward A and T, with 70.5% of A + T content (A 38.8%, T 31.7%, C 16.6%, and G 12.9%). Gene order was conserved and identical to most other previously sequenced Libellulidae dragonflies. Most PCGs of *N. fulvia* have the conventional start codons ATN (six ATG, three ATT, and two ATC), with the exception of *cox1* and *nad1* (TTG). Except for four PCGs (*cox1*, *cox2*, *cox3*, and *nad5*) end with the incomplete stop codon T––, all other PCGs terminated with the stop codon TAA or TAG. Phylogenetic analysis showed that *N. fulvia* got together with *Tramea virginia* with high support value. Libellulidae had a close relationship with Corduliidae, the relationships ((*Hydrobasileus* + *Brachythemis*) + (*Orthetrum* + (*Acisoma* + (*Neurothemis* + *Tramea*)))) were supported in Libellulidae.

*Neurothemis* (Red dragonflies) is a Libelluliae member commonly found in drains, ditches, shallow streams, and paddy fields (Jisha Krishnan and Sebastian 2016). Most of the female members of *Neurothemis* exhibit female-limited polymorphism with a clear difference in the wing and body coloration compared to males (Tabugo et al. 2014; Jisha Krishnan and Sebastian 2016). The fulvous forest skimmer, *Neurothemis fulvia*, is a medium-sized rusty dragonfly with transparent wing tips. Male is reddish brown whereas the female is paler brown in color. It breeds in marshes associated with forest streams and rivers.

Specimens of *N. fulvia* were collected from Jian City, Jiangxi Province, China (26°36′N, 114°8′E, July 2018) and were stored in Entomological Museum of Gannan Normal University (Accession number GNU-ENF02). Total genomic DNA was extracted from tissues using DNeasy DNA Extraction kit (Qiagen, Hilden, Germany). The mitogenome sequence of *N. fulvia* was generated using Illumina HiSeq 2500 Sequencing System (Illumina, San Diego, CA). A total of 30.8 million reads were generated and had been deposited in the NCBI Sequence Read Archive (SRA) with accession number SRR12805489. Raw reads were assembled using MITObim version 1.7 (Hahn et al. 2013). By comparison with the homologous sequences of other Libellulidae species from GenBank, the mitogenome of *N. fulvia* was annotated using software GENEIOUS R11 (Biomatters Ltd., Auckland, New Zealand).

The complete mitogenome of *N. fulvia* is 15,459 bp in length (GenBank accession no. MT371046), and containing the typical set of 13 protein-coding (PCGs), 2 ribosomal RNA (rRNA), and 22 transfer RNA (tRNA) genes, and one non-coding AT-rich region. The nucleotide composition of the mitogenome was biased toward A and T, with 70.5% of A + T content (A 38.8%, T 31.7%, C 16.6%, and G 12.9%). Gene order was conserved and identical to most other previously sequenced Libellulidae dragonflies (Tang et al. 2014; Yong et al. 2016; Yu et al. 2016; Jeong et al. 2018; Guan et al. 2019). Most PCGs of *N. fulvia* have the conventional start codons ATN (six ATG, three ATT, and two ATC), with the exception of *cox1* and *nad1* (TTG). Except for four PCGs (*cox1*, *cox2*, *cox3*, and *nad5*) end with the incomplete stop codon T−, all other PCGs terminated with the stop codon TAA or TAG. Two rRNA genes (*rrnL* and *rrnS*) locate at *trnL1*/*trnV* and *trnV*/control region, respectively. The lengths of *rrnL* and *rrnS* in *N. fulvia* are 1281 and 749 bp, with the AT contents of 74.9% and 72.4%, respectively. All 22 tRNA genes were predicated in this study and vary from 63 bp (*trnT*) to 71 bp (*trnK*).

Phylogenetic analysis was performed based on the nucleotide sequences of 13 PCGs from 20 Odonata species. Phylogenetic tree was constructed through raxmlGUI version 1.5 (Silvestro and Michalak 2012). Results showed that the new sequenced species *N. fulvia* got together with *Tramea virginia* with high support value (Figure 1). Libellulidae had a close relationship with Corduliidae, the relationships ((*Hydrobasileus* + *Brachythemis*) + (*Orthetrum* + (*Acisoma* + (*Neurothemis* + *Tramea*)))) were supported in Libellulidae. In conclusion, the mitogenome of *N. fulvia* is sequenced in this study and can provide essential and important DNA molecular data for further phylogenetic and evolutionary analysis of Libellulidae.

**Figure 1.**
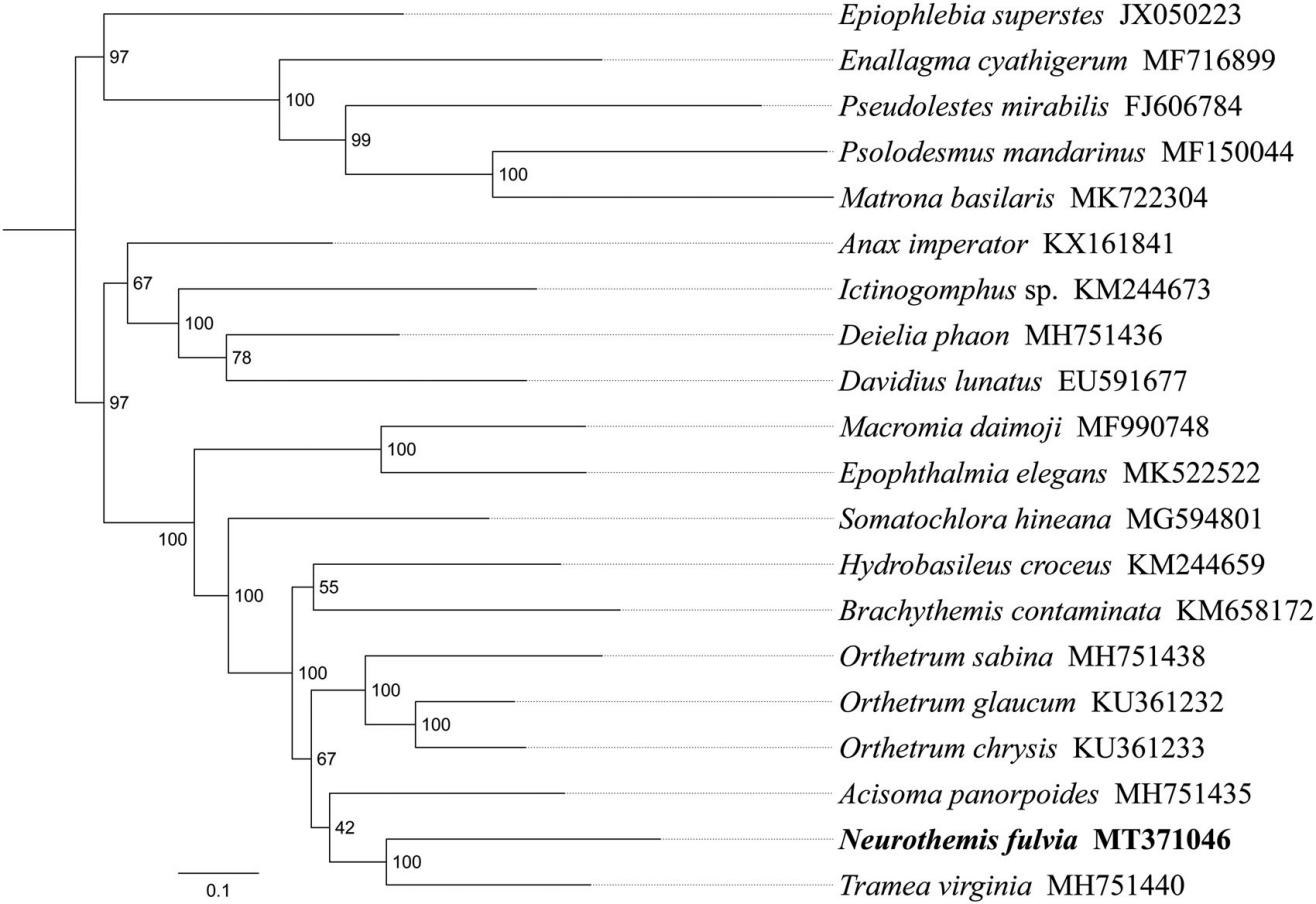
Phylogenetic relationships based on the 13 mitochondrial protein-coding genes sequences inferred from RaxML. Numbers on branches are Bootstrap support values (BS).

## Data Availability

The data that support the findings of this study are openly available in NCBI (National Center for Biotechnology Information) at https://www.ncbi.nlm.nih.gov/, reference number MT371046, SRR12805489.
